# From the perception of a cluster of cases of children with microcephaly to congenital Zika syndrome in Brazil: the lessons we have learned and the challenges that lie ahead of us

**DOI:** 10.1186/s40409-017-0107-x

**Published:** 2017-03-20

**Authors:** Erlane Marques Ribeiro, Thayse Figueiredo Lopes, Sáile Cavalcante Kerbage, André Luis Santos Pessoa, Luciano Pamplona de Góes Cavalcanti

**Affiliations:** 1 Albert Sabin Children’s Hospital, Fortaleza, CE Brazil; 2 Christus University Center, Fortaleza, CE Brazil; 3 Department of Community Health, School of Medicine, Federal University of Ceará, Rua Prof. Costa Mendes, 1608, 5 andar, Fortaleza, CE CEP 60430-140 Brazil

Dear Editor of JVATiTD,

A little more than a year ago, physicians and researchers from the northeastern region of Brazil raised the hypothesis of an association between microcephaly cases in newborns and a possible Zika virus infection in their mothers during pregnancy. Common phenotypic features called the attention of the discerning eyes of geneticists, already used to this type of observation [1]. In those cases, records of exanthematous disease during pregnancy were found in the anamnesis. Moreover, radiology images revealed findings that although resembled some other TORCH, they had their particularities in common. Initially, a recurrent pattern in computerized tomography of the skulls was described, which led physicians to classify the set of findings as the emergence of a new disease [2].

For professionals who were experiencing this reality in the Northeast, there was no doubt that the puzzle was being solved. Chronology, clinical history and findings, all of these suggested that the exanthematous disease reported by those women during pregnancy was related to the microcephaly of their babies [3]. Although there was a strong distrust concerning causal relationship and at that time no scientific basis to corroborate the hypothesis, for many physicians Zika virus was underestimated, an apparently self-limited disease with discrete symptoms that people often overlooked.

Despite emerging evidence, several researchers were skeptical. They did not even consider the possibility of teratogeny provoked by an epidemic of Zika virus disease. In this scenario of panic and uncertainties – permeated by mismatched information among physicians, researchers, journalists and the general population – the scientific community was divided, living moments of collective hysteria. Misleading information on social networks and sensationalist news made this moment even more complicated and moving. It also should be noted that it all occurred in a very troubled political and economic moment in Brazil.

At this first moment, there were no sources in the literature to provide information on necessary measures or even follow-up protocols. Nothing could backed up the physicians and guide them in the investigation. There were distressed parents waiting for a diagnosis or explanation, full of doubts that health professionals could not clarify. At that moment, several questions were repeated: “What is the problem of our baby?”, “Will he be fine?”, “Will he survive?”, “Are you sure it was Zika?” and “What do I do to make him better?”.

Despite the feeling of powerlessness in face of the unknown, physicians in charge had to be sincere and to use their experiences with similar pathologies to manage the cases of these babies. They requested many tests to rule out other possible causes and referred children to many specialists [4, 5]. As therapeutic management, they indicated early stimulation with physiotherapy, speech therapy and occupational therapy in order to avoid complications that could lead to death. They did so in the hope that this work would stimulate the incredible intrinsic neuronal plasticity of children and could bring to these patients a better perspective of development, despite the severe neurological damage already established.

The news about “the new disease” spread, and soon, physicians, researchers and other health Brazilian professionals became harassed by the international scientific community. They wanted information about children, about pregnant women, blood samples, umbilical cord samples, placental tissue, among other data. There was an intensification of research, and the more it advanced, the more the evidence of the relationship between the infection and the pathology of these babies became irrefutable.

With the dissemination of information in digital media, social networks, besides scientific media, the society became aware of the possible risk it was exposed to. The fear in face of the possibility that a planned and desired child could be born with a serious illness had taken hold of many families, and the social repercussion of it was immense. Countless families with women of childbearing age avoided trips to the Northeast, compromising tourism in the region. Brazilian women from more wealthy families decided, temporarily, to disconnect from their families during pregnancy and move to other countries that did not offer this risk to their children. Moreover, for other women, the dream and planning of motherhood was simply postponed indefinitely [6]. In that beginning, hypothesis related to the syndrome ranged from conspiracy theory to fearful transmission forms. This made pregnant women and relatives paranoid and some of them have even considered the possibility of abortion.

Why did this happen? It is true that historically there is lack of information and resources to combat the transmitter mosquito. It was always easier to repeat that governments were not acting properly. There were always other people to be blamed, even neighbors, in that moment of desperation and distress, in which *Aedes aegypti* mosquitoes could be found in almost every home.

A few months after the appearance of the first cases, the Brazilian Ministry of Health, with the support of some medical societies, added efforts in a task force to produce and release information weekly (even daily at some moments) that could guide the necessary decisions. At that time, the occurrence of serious cases of microcephaly with characteristics of a “new disease"” was reported. In this regard, the challenge was to review and update appropriate surveillance protocols that at various moments were questioned. The search was always for a greater sensitivity of the system, in an attempt of not causing unnecessary panic in pregnant women under the suspicion of having the disease.

Initially, the surveillance was carried out focusing on microcephaly cases, but there were other questions: “How to identify milder cases?”, “How can we identify cases with neurological damage, but no microcephaly?”, “Can a childcarer detect the slightest sign of developmental delay?”, “Wouldn’t we be wasting time stimulating these children?”, “Is neurological damage the only one possible?”, “How did the virus interfere with the immune system of these mothers and babies?” and “How long did the virus stay alive in the baby’s body?” To answer these, there was a basic need to confirm the cases in a laboratory. The aggravating factor is that the Zika epidemic in the Northeast occurred simultaneously with those of dengue and chikungunya, and laboratory kits for diagnosis did not show enough sensitivity and specificity.

Just over a year after the first zika virus epidemic in northeastern Brazil, there are still more doubts than assurances. We are no longer talking about a single disease transmitted by a unique vector, but also by sexual contact and possible transmission by saliva, transfusion, breast milk, sweat and tears [7–12]. The Zika virus infection that initially appeared to be harmless is now of devastating consequences and many doubts remain [13, 14]. What is the real cause for the higher occurrence in northeastern Brazil? Is it related to low coverage for yellow fever vaccine? Is it associated with the high prevalence of dengue in this region? With poverty? With lack of sanitation? More important than all is to know which measures should be taken by the caregivers and health care professionals responsible for these pregnant women and their children.

Among these other important questions, one remains pivotal: what tests should be performed? Should the tests suggested in 2015 be applied nowadays, one year after the first management experiences of these patients? It seems not to be the case [15].

We are no longer talking about cases of microcephaly probably associated with Zika virus, but rather with a new Zika congenital syndrome. In the case of pregnant women in the first prenatal visit, should we still recommend only TORCH or can we work with the expression ZTORCH? Or would it be TORCHZ?

Physicians have performed symptomatic treatment for children with congenital Zika syndrome for dysphagia, orthopedic deformities and seizures, consequences from a neurological damage. Is there any therapy that can be used to prevent neurological sequelae? If the infection is active only in the prenatal period, it seems that there is no such treatment.

With a little more than a year of experience in the management of these babies, new challenges have arisen. It is worth noting those of handling adequately seizures. There are light and focal cases that most professionals cannot even recognize them until the development of next episodes. Is this one of the possible causes of the excessive crying of the affected ones? Will the drugs used for seizures in other causes be effective in these cases? Unfortunately, tests such as electroencephalography and some medications for seizure are not available for everyone.

Which path should we follow? The need for further research and assistance for those already affected by the congenital Zika syndrome epidemic remain a current topic, subject of wide discussion and incessant researches. We hope this episode will serve as warning, so that in the future the same mistakes do not occur, making us more able to interfere more quickly in the identification, prevention and treatment of new teratogens.

## Abbreviations

TORCH: Acronym for toxoplasmosis other infections (syphilis, varicella-zoster, parvovirus b19), rubella, cytomegalovirus and herpes infections
